# Differences in chronic musculoskeletal pain among male and female Brazilian university professors*

**DOI:** 10.1590/1980-220X-REEUSP-2025-0263en

**Published:** 2026-01-23

**Authors:** Mônica Jordão de Souza Pinto, Inmaculada Failde, Priscilla Hortense

**Affiliations:** 1 Universidade Federal de São Carlos, Programa de Pós-graduação em Enfermagem, São Carlos, SP, Brazil.; 2 Universidad de Cádiz, Medicina Preventiva y Salud Pública, Cadiz, Andalucía, España.; 3 Universidade Federal de São Carlos, Departamento de Enfermagem, São Carlos, SP, Brazil.

**Keywords:** Chronic Pain, Faculty, Sex Characteristics, Self Efficacy, Dor Crônica, Docentes, Caracteres Sexuais, Autoeficácia

## Abstract

**Objective:**

To compare sociodemographic, clinical, pain, work, and self-efficacy aspects between male and female Brazilian federal university professors with chronic musculoskeletal pain.

**Methods:**

This cross-sectional study included 974 university professors with chronic musculoskeletal pain. Data collected: an e-survey between January and May 2023. Variables: sociodemographic and clinical characteristics, pain intensity, self-efficacy in chronic pain, and work productivity loss. Statistical analyses used t-tests, Mann-Whitney tests, and regression models.

**Results:**

Women reported 27% more body areas with pain and 10% higher pain intensity than men. Continuous pain medication use was 53% more frequent among women, who also engaged in 49% more rehabilitation treatments. Women scored lower in pain management self-efficacy and experienced greater productivity loss. The most common pain locations for women were the suprascapular region, while men reported lower back pain. Women used more analgesics and anticonvulsants, and men had higher rates of overweight and obesity.

**Conclusions:**

Women experience more severe and widespread chronic musculoskeletal pain, use more medications and rehabilitation, and have lower self-efficacy and higher productivity loss compared to men.

## RESUMO

**Objetivo:**

Comparar aspectos sociodemográficos, clínicos, de dor, de trabalho e de autoeficácia entre professores universitários federais brasileiros do sexo masculino e feminino com dor musculoesquelética crônica.

**Métodos:**

Este estudo transversal incluiu 974 professores universitários com dor musculoesquelética crônica. Dados coletados: pesquisa online realizada entre janeiro e maio de 2023. Variáveis: características sociodemográficas e clínicas, intensidade da dor, autoeficácia no controle da dor crônica e perda de produtividade no trabalho. As análises estatísticas utilizaram testes t, testes de Mann-Whitney e modelos de regressão.

**Resultados:**

As mulheres relataram 27% mais áreas do corpo com dor e uma intensidade de dor 10% maior do que os homens. O uso contínuo de analgésicos foi 53% mais frequente entre as mulheres, que também realizaram 49% mais tratamentos de reabilitação. As mulheres obtiveram pontuações mais baixas em autoeficácia no controle da dor e apresentaram maior perda de produtividade. A região de dor mais comum entre as mulheres foi a região supraescapular, enquanto os homens relataram dor na região lombar. As mulheres usaram mais analgésicos e anticonvulsivantes, e os homens apresentaram taxas mais altas de sobrepeso e obesidade.

**Conclusões:**

As mulheres sofrem de dores musculoesqueléticas crônicas mais intensas e generalizadas, utilizam mais medicamentos e reabilitação, e apresentam menor autoeficácia e maior perda de produtividade em comparação aos homens.

## INTRODUCTION

Chronic pain represents a significant public health challenge and understanding its prevalence contributes to the development of appropriate healthcare strategies. Recent studies conducted in the United States and Spain assessed the prevalence of chronic pain, reporting a prevalence of 20.5% among adults in the United States^(1)^ and 25.9% among adults and older adults in Spain^(2)^. In Brazil, the prevalence of chronic pain was identified in a systematic review with meta-analysis in the general adult population, ranging from 23.02% to 41.4%^(3)^.

Among the different types of chronic pain, chronic musculoskeletal pain stands out due to its high prevalence, making it a major health concern for researchers, clinicians, and employers worldwide. A study conducted in several European countries with 61,157 individuals from the general population over 50 years of age showed that the prevalence of chronic musculoskeletal pain was 35.7%, ranging from 18.6% in Switzerland to 45.6% in France^(4)^. The prevalence in low- and middle-income countries was 26%, 39%, and 86% in adults, older adults, and workers, respectively^(5)^.

Sex constitutes a relevant factor regarding the disparities in the prevalence and characteristics of individuals with chronic musculoskeletal pain. The rates of this type of pain are higher among women^(2,4,6,7,8,9,10)^, who have greater intensity, frequency, number of areas of the body affected, and duration compared to men^(6–7,9–10)^. In addition, women are also more affected by diseases that cause pain, such as fibromyalgia, temporomandibular disorders, osteoarthritis and rheumatoid arthritis, as well as neck pain and low back pain^(6,10)^.

The world of work is a prominent setting for investigating sexual disparities in chronic musculoskeletal pain given its prevalence among workers^(5)^. This context is even more relevant for investigation as it allows the comparison between men and women, considering that, in addition to formal work, women accumulate other roles, increasing their overload and exposure to the risk of chronic musculoskeletal pain.

Professors are exposed to significant risk factors for the development of musculoskeletal disorders and, consequently, chronic musculoskeletal pain. These factors are related to the nature of physical teaching work, including standing and/or sitting for extended periods in inadequate postures, bending over desks to read or assess students’ work, and carrying and transporting heavy books or classroom equipment^(11)^. In Brasil, university professors represent an important group of workers with high workload, including teaching, research, extension and management. Therefore, identifying health demands in this group to know more about sexual differences in chronic musculoskeletal pain can provide knowledge to create treatment protocols adapted to the identified needs^(6,7,10)^.

An analysis of these differences, both in terms of clinical aspects and those related to work and pain management, can provide comprehensive and fundamental understanding to outline the involvement profile and be crucial evidence for clinicians and employing institutions develop specific management strategies. Thus, the present study contributes to the knowledge arising from research in humans with chronic pain, as it differs from many others which have evaluated sex differences in pain in animal models or induced in healthy volunteers^(12)^.

Therefore, this study aims to compare men and women regarding sociodemographic, clinical, pain, work and self-efficacy aspects in a sample of professors from Brazilian federal public universities who present chronic musculoskeletal pain.

## METHODS

### Design

This study is an analytical and cross-sectional investigation^(13)^ guided by the STROBE guidelines.

### Local

The study was conducted in the 68 active Brazilian federal universities.

### Sample Definition

To recruit participants, we used a convenience sampling approach. Monthly invitations to participate were sent to approximately 9,000 randomly selected emails on university websites, including the personal emails of professors and the emails of secretariats with requests to disseminate the survey. These invitations were not specifically targeted at individuals with musculoskeletal pain but aimed at the general university population. To achieve the objectives of this study, only professors who responded positively whether or not they had musculoskeletal pain for 6 months or more, were included. To assist in this response, the concept of musculoskeletal pain was attatched to the question and participants were requested to indicate for how long they had pain, so we were able to include only those who reported pain for 6 months or more. We obtained 1436 responses, and 462 professors were excluded because they did not report having chronic musculoskeletal pain.

### Population and Selection Criteria

The inclusion criteria were being a professor, in a statutory employment regime (civil servant with work stability), and self-reporting chronic musculoskeletal pain for six months or more. Professors appointed to their position less than one year before the date of data collection, those who were away from their work activities in the last two weeks after receiving the invitation, retirees, and those with chronic oncological pain were excluded.

### Data Collection and Studied Variables

Data collection was conducted virtually (e-survey) between January and May 2023. The variables investigated for comparison between sexes were self-reported and included age; body mass index (BMI); number of body regions with pain; comorbidities; pain location, intensity and frequency; pharmacological and non-pharmacological treatments for pain; self-efficacy; work data (another job, overtime - number of hours worked beyond the employment contract, satisfaction); and loss of productivity at work. It is important to highlight that this study only focused on sex as a biological variable and did not investigate the influence of gender, which refers to roles, behaviors and social identities.

Pain intensity was measured using an 11-point numerical scale and was categorized as follows: a value of zero represents no pain, a range of 1 to 3 indicates mild pain, a range of 4 to 6 indicates moderate pain, and a range of 7 to 10 indicates severe pain^(14)^, and the treatments evaluated included pharmacological measures (name of the medications used, which were classified by pharmacological class) and non-pharmacological measures (rehabilitation), including Pilates, swimming, water aerobics, acupuncture, meditation, physiotherapy, overall postural re-education, psychotherapy, nutritional monitoring, physical exercises with a physical educator, monitoring with a doctor, pain support group and others, in addition to the completion time in months.

Subsequently, the Chronic Pain Self-Efficacy Scale (CPSS), validated in Brazil^(15)^, was used to measure self-efficacy for chronic pain, with 22 questions distributed in three domains: self-efficacy for pain management, self-efficacy for physical functioning and self-efficacy for coping with symptoms. Scores range from 30 to 300, and the higher the score, the greater the level of self-efficacy for chronic pain.

Loss of productivity was estimated using the Work Limitations Questionnaire (WLQ-25), validated in Brazil^(16)^, and a confidentiality agreement was signed.

### Data Analysis and Treatment

Data were described by absolute and percentage frequencies (qualitative variables) and by central tendency and dispersion measures (mean, standard deviation, minimum, median and maximum) for quantitative variables, stratified by sex.

Comparisons of continuous quantitative variables between sexes were performed using the Student’s t-test or Mann-Whitney test, according to normality (verified by histogram and Kolmogorov-Smirnov test). A negative-binomial regression model and logarithmic link function were used for discrete quantitative variables (number of regions with pain, average pain intensity and number of medications in continuous use), calculating the relative increase or reduction in the mean by AR(>β) = [exp(>β) – 1]*100%. Categorical variables with more than two response levels were analyzed by the chi-squared test, while binary variables were analyzed by a log-binomial regression model with estimation of the prevalence ratio. All analyses were performed using SAS 9.4 software with a significance level of 5%, and graphs were generated using R Studio software (version 2023.12.0.369).

### Ethical Aspects

The study was approved by the Research Ethics Committee of the Universidade Federal de São Carlos (opinion no. 5.741.976) and all participants agreed to participate by signing the Informed Consent Form in digital format.

## RESULTS

The study included 974 university professors with chronic musculoskeletal pain from all regions of Brazil, 622 of whom were women (63.8%) and 352 (36.1%) were men, with little variation in mean ages. The frequency of comorbidities was similar between the sexes, and men had a higher rate of overweight and obesity regarding BMI (Table 1).

**Table 1:** Comparison between male and female federal university professors with chronic musculoskeletal pain regarding age, clinical characteristics, pain and its management, and chronic pain self-efficacy scale (CPSS) – Brazil, 2023.

Variable	Female (n = 622)	Male (n = 352)	P-value	Effect (95%CI) Fem. vs Male
**Age**			0.75^1^	ED: 0.19 (–0.99;1.37)
Mean (SD)	48.11 (8.93)	47.92 (9.1)		
Median (Min-Max)	47 (29–73)	47 (21–74)		
**Comorbidities**				
No	378 (60.77%)	209 (59.38%)		
Yes	244 (39.23%)	143 (40.63%)	0.67^4^	PR: 0.97 (0.82;1.13)
**BMI**			<0.01^3^	
Underweight	11 (1.77%)	1 (0.28%)		
Normal weight	283 (45.5%)	106 (30.11%)		
Overweight	210 (33.76%)	161 (45.74%)		
Obese	118 (18.97%)	84 (23.86%)		
**Number of body areas feeling pain**			<0.01^5^	RD: 1.27 (1.17;1.39)
Mean (SD)	4.38 (2.98)	3.44 (2.31)		
Median (Min-Max)	4 (0–17)	3 (0–17)		
**Pain intensity**			**<0.01** ^5^	RD: 1.10 (1.04;1.17)
Mean (SD)	5.97 (2.13)	5.41 (2.02)		
Median (Min-Max)	6 (0–10)	6 (0–10)		
**Weekly pain frequency**			0.01^3^	
1 time	38 (6.11%)	22 (6.25%)		
2 times	70 (11.25%)	50 (14.2%)		
3 times	81 (13.02%)	69 (19.6%)		
4 times	72 (11.58%)	42 (11.93%)		
5 times	98 (15.76%)	44 (12.5%)		
6 times	39 (6.27%)	9 (2.56%)		
7 times	224 (36.01%)	116 (32.95%)		
**Continuously use pain medications**				
No	382 (61.41%)	263 (74.72%)		
Yes	240 (38.59%)	89 (25.28%)	<0.01^4^	PR: 1.53 (1.24;1.87)
**Number of medications used continuously for pain**			**<0.01** ^5^	RD: 1.68 (1.32;2.14)
Mean (SD)	0.63 (0.97)	0.38 (0.75)		
Median (Min-Max)	0 (0–5)	0 (0–4)		
**Intermittent pain medication use**				
No	203 (32.64%)	123 (34.94%)		
Yes	419 (67.36%)	229 (65.06%)	0.47^4^	PR: 1.04 (0.94;1.14)
**Rehabilitation for pain**				
No	265 (42.6%)	216 (61.36%)		
Yes	357 (57.4%)	136 (38.64%)	**<0.01** ^4^	PR: 1.49 (1.28;1.72)
**Self-efficacy for pain management**			**<0.01** ^1^	ED: –4.04 (–7.02;–1.06)
Mean (SD)	57.86 (22.67)	61.9 (22.97)		
Median (Min-Max)	58 (10–100)	64 (10–100)		
**Self-efficacy for physical functioning**			<0.01^2^	
Mean (SD)	70.34 (26.06)	75.06 (26.71)		
Median (Min-Max)	76.67 (0–100)	80 (0–100)		
**Self-efficacy for coping with symptoms**			**<0.01** ^1^	ED: –4.83 (–7.65;–2.02)
Mean (SD)	57.03 (21.25)	61.86 (21.97)		
Median (Min-Max)	57.5 (0–100)	63.75 (10-100)		
**Total self-efficacy score**			**<0.01** ^1^	ED: –13.59 (–21.9;–5.28)
Mean (SD)	185.22 (62.82)	198.81 (64.57)		
Median (Min-Max)	193.53 (29.86–300)	209.42 (30–300)		

^1^Student’s t-test; ED: Estimated difference.

^2^Mann-Whitney test.

^3^Chi-squared test.

^4^ Log-binomial regression; PR: Prevalence ratio.

^5^ Negative binomial regression with logarithmic link function; RD: Relative difference.

Regarding pain, the number of body regions with pain in women is 27% higher compared to men and they experience 10% more intense pain than men (Table 1). The most reported region among women professors is the suprascapular region, while for men is the lower back region (Table 2). In addition, other pain sites frequently reported by both sexes were the cervical region and shoulders.

**Table 2:** Comparison between male and female federal university professors with chronic musculoskeletal pain regarding their body location – Brazil, 2023.

Pain location	Female (n = 622)	Male (n = 352)	p-Value^1^	PR (IC95%)
Suprascapular	370 (59.49%)	130 (36.93%)	<0.01	1.61 (1.38;1.87)
Lower back	325 (52.25%)	178 (50.57%)	0.62	1.03 (0.91;1.17)
Cervical	273 (43.89%)	118 (33.52%)	<0.01	1.31 (1.1;1.55)
Shoulders	265 (42.6%)	114 (32.39%)	<0.01	1.32 (1.1;1.57)
Hip	232 (37.3%)	89 (25.28%)	<0.01	1.48 (1.2;1.81)
Knees	173 (27.81%)	101 (28.69%)	0.77	0.97 (0.79;1.19)
Wrists	154 (24.76%)	53 (15.06%)	<0.01	1.64 (1.24;2.18)
Upper back	143 (22.99%)	72 (20.45%)	0.36	1.12 (0.87;1.45)
Hands	135 (21.7%)	44 (12.5%)	<0.01	1.74 (1.27;2.38)
Feet	117 (18.81%)	53 (15.06%)	0.14	1.25 (0.93;1.68)
Mid back	113 (18.17%)	69 (19.6%)	0.58	0.93 (0.71;1.21)
Elbows	99 (15.92%)	44 (12.5%)	<0.01	1.32 (1.1;1.57)
Ankles	90 (14.47%)	38 (10.8%)	0.11	1.34 (0.94;1.91)
Forearms	69 (11.09%)	18 (5.11%)	<0.01	2.17 (1.31;3.58)
Thighs	60 (9.65%)	38 (10.8%)	0.57	0.89 (0.61;1.31)
Legs	54 (8.68%)	34 (9.66%)	0.61	0.9 (0.6;1.35)
Arms	52 (8.36%)	17 (4.83%)	0.04	1.73 (1.02;2.95)

^1^ Log-binomial regression;

PR: Prevalence ratio.

The frequency of continuous pain medication use in women is 53% higher than in men (Table 1) and the most used medications by both sexes are anti-inflammatories and muscle relaxants. The use of analgesics and anticonvulsants is higher among women (Table 3).

The frequency of rehabilitation for pain is 49% higher among women, who undergo an average of 1.76 types of rehabilitation, while men undergo 1.02 (Table 1). Physiotherapy is the most sought-after type, followed by Pilates. Other frequent interventions were medical follow-up, acupuncture, and physical exercises with a physical education professional (Table 3).

**Table 3:** Comparison between male and female federal university professors regarding pharmacological and non-pharmacological measures (rehabilitation) used in pain management – Brazil, 2023.

Treatments for pain	Female (n = 613)	Male (n = 349)	p-Value	PR (IC 95%) Fem. vs Mal.
**Continuous-use medications**				
Anti-inflammatories	103 (16.8%)	47 (13.47%)	0.17^1^	1.25 (0.91;1.72)
Muscle relaxants	90 (14.68%)	27 (7.74%)	<0.01^1^	1.9 (1.26;2.86)
Analgesics	56 (9.14%)	17 (4.87%)	0.02^1^	1.88 (1.11;3.18)
Anticonvulsants	38 (6.2%)	14 (4.01%)	0.15^1^	1.55 (0.85;2.81)
Antidepressants	35 (5.71%)	4 (1.15%)	<0.01^1^	4.98 (1.79;13.9)
Narcotic analgesics	33 (5.38%)	11 (3.15%)	0.12^1^	1.71 (0.87;3.34)
Others	17 (2.77%)	6 (1.72%)	0.31^1^	1.61 (0.64;4.05)
Systemic glucocorticoids	14 (2.28%)	5 (1.43%)	0.37^1^	1.59 (0.58;4.39)
**Rehabilitation**				
Physiotherapy	229 (36.82%)	83 (23.58%)	<0.01^1^	1.56 (1.26;1.93)
Pilates	211 (33.92%)	65 (18.47%)	<0.01^1^	1.84 (1.44;2.35)
Monitoring with a doctor	117 (18.81%)	30 (8.52%)	<0.01^1^	2.21 (1.51;3.22)
Acupuncture	112 (18.01%)	31 (8.81%)	<0.01^1^	2.04 (1.4;298)
Physical exercises with a physical educator	89 (14.31%)	30 (8.52%)	<0.01^1^	1.68 (1.13;2.49)
Other	62 (9.97%)	32 (9.09%)	0.66^1^	1.1 (0.73;1.65)
Overall postural re-education	60 (9.65%)	9 (2.56%)	<0.01^1^	3.77 (1.9;7.51)
Psychotherapy	53 (8.52%)	14 (3.98%)	<0.01^1^	2.14(1.21;3.8)
Nutritional monitoring	46 (7.4%)	16 (4.55%)	0.08^1^	1.63(0.94;2.83)
Water aerobics	40 (6.43%)	10 (2.84%)	0.02^1^	2.26(1.15;4.47)
Swimming	36 (5.79%)	26 (7.39%)	0.33^1^	0.78(0.48;1.28)
Meditation	35 (5.63%)	12 (3.41%)	0.13^1^	1.65(0.87;3.14)
Pain support group	3 (0.48%)	0 (0%)	0.56^3^	–
**Number of rehabilitation activities**			<0.01^2^	–
Mean (SD)	1.76 (1.96)	1.02 (1.62)		
Median (Min-Max)	1 (0–10)	0 (0–10)		

^1^ Log-binomial regression;

PR: Prevalence ratio.

^2^ Mann-Whitney test.

^3^ Fisher’s exact test.

Women scored 13.59 points lower than men regarding the total self-efficacy score. In addition, women scored 4 and 4.8 points lower than men in the self-efficacy pain management and coping with symptoms domains, respectively. There was no statistically significant difference between the sexes in the number of hours worked beyond the weekly workday. Women professors took 16% fewer breaks during the workday compared to men professors, in addition to presenting an average loss of productivity at work which was 1.22% higher (Table 4).

**Table 4:** Comparison between male and female federal university professors with chronic musculoskeletal pain regarding work-related data and loss of productivity at work – Brazil, 2023.

Variable	Female (n = 622)	Male (n = 352)	P-value	Effect (95%CI) Fem. vs Mal.
**span class="Bold">Working hours greater than the weekly working hours**			0.183	PR: 1.05 (0.98;1.14)
No	145 (23.31%)	96 (27.27%)		
Yes	477 (76.69%)	256 (72.73%)		
**Number of additional hours worked per week**			0.472	
Less than 1 hour/week	2 (0.42%)	0 (0%)		
From 1 to 5 hours/week	164 (34.38%)	90 (35.16%)		
From 6 to 10 hours/week	166 (34.8%)	98 (38.28%)		
More than 10 hours/week	145 (30.4%)	68 (26.56%)		
**Rest break during the workday**			**<0.014**	PR: 0.84 (0.76;0.93)
No	261 (41.96%)	109 (30.97%)		
Yes	361 (58.04%)	243 (69.03%)		
**Work satisfaction**			0.743	PR: 0.99 (0.95;1.03)
No	57 (9.18%)	30 (8.55%)		
Yes	564 (90.82%)	321 (91.45%)		
**Loss of productivity at work (percentage)**			**<0.011**	ED: 1.22 (0.57;1.88)
Mean (SD)	8.27 (5.03)	7.05 (4.9)		
Median (Min-Max)	7.51 (0–24.08)	6.2 (0–21.84)		

^1^Student’s t-test; ED: Estimated difference.

^2^Chi-squared test.

^3^Log-binomial regression. PR: Prevalence ratio.

An unadjusted log-binomial regression model with each independent variable analyzed individually was used for quantitative independent variables, the prevalence ratio was estimated for each 1-unit increase in the independent variable. In women, increasing age, the number of body regions with pain, a greater number of continuous pain medications, dissatisfaction with teaching, and greater loss of productivity are associated with a higher prevalence of moderate or severe pain (score ≥ 4).

Furthermore, a higher self-efficacy score is associated with a lower prevalence of moderate or severe pain (score ≥ 4). In men, greater productivity loss is associated with a higher prevalence of moderate or severe pain (on average, for every 1-point increase in productivity loss, the prevalence of moderate or severe pain increases by 1.3%) and a higher score self-efficacy is associated with a lower prevalence of moderate or severe pain. However, in adjusted analyses with all variables, no associations were found. Likewise, there were no associations in a second adjusted analysis including as independent variables only those with a p value <0.20 in the crude analyses.

## DISCUSSION

The results of this study show the sexual differences in chronic musculoskeletal pain among university professors, and these differences are evidenced by the higher prevalence of this type of pain among women. Women also presented greater pain intensity and a greater number of affected areas, greater frequency of medication use and rehabilitation treatments for pain, lower general self-efficacy index and self-efficacy for pain management and coping with symptoms, fewer breaks during the workday, and greater loss of productivity at work compared to men.

The findings of the present study show what other studies have found regarding the higher prevalence of chronic pain among women; such studies have shown that genetic, anatomical, physiological, neuronal, hormonal, psychological, social and cultural aspects influence and cause differences in the perception, modulation and expression of pain between the women and men^(7,10)^, and that such differences include communication, attitudes and pain behavior.

It should be highlighted that sexual differences in pain perception are strongly impacted by psychosocial and cultural factors that can act from birth, through childhood, and into adulthood^(17-18)^. These factors are possibly linked to the way society treats pain in boys and girls, as well as in women and men. Psychosocial variables play an important role in developing and maintaining pain, in addition to modulating treatment outcomes^(17)^. The psychosocial variables which have been shown to play this role are negative affect, childhood trauma, and social support, as well as pain catastrophizing, self-efficacy in dealing with pain, and coping with pain^(18)^.

The various roles assumed by women in today’s society, the accumulated responsibilities, the demands for optimal performance in the roles of wife, mother, “home” manager, and worker are important aspects which shape pain expression and modulate nociceptive stimulus, explaining the results of greater prevalence, intensity and number of areas of the body with pain. Moreover, there is the possible lack of social support for women in their roles, which generates physical and emotional overload and contributes to pain worsening^(18)^.

In addition to these issues, it is important to emphasize that the vast majority of public university professors in Brazil have an exclusive work schedule of 40 hours per week, and are responsible for teaching, research, extension and university management activities. Added to all the other responsibilities assumed for women, this schedule can lead to several consequences, including physical and mental exhaustion, accentuating and aggravating the condition of chronic pain and other health conditions^(19)^.

Regarding the pain location, the cervical and shoulder regions were common areas of pain in both sexes. The literature indicates that these areas are frequently reported as a focus of pain in workers who perform their activities in front of computers and electronic devices for hours and hours, and often without the appropriate ergonomic adjustments of the workstations required to prevent pain^(20)^. This especially applies to the professor category in Brazil, who mostly perform their tasks in a sitting position, such as preparing classes for undergraduate and graduate students, correcting undergraduate research scholarship , master’s and doctoral studies, reading and scientific research activities, student management, among others.

The frequency of comorbidities was similar between the sexes, suggesting that despite the disparities found in the intensity and number of pain regions, women and men presented a similar health status in relation to other clinical conditions. This result is relevant, as it demonstrates that variations in chronic musculoskeletal pain are not associated with a greater number of comorbidities between the groups. However, it is important to emphasize that although professors of both sexes have an average age of 48 years, around 40% of each group have comorbidities. This index is concerning when compared to the Brazilian population aged 45 to 54 years without pain, whose morbidity prevalence is 34.7% for hypertension, 10.4% for diabetes mellitus, and 11.8% for depression^(21)^.

Additionally, individuals with chronic pain have a higher prevalence of comorbidities, and pain can act as a risk factor for the worsening of these conditions, creating a vicious cycle that increases the disease burden, resulting in significantly higher rates of medical consultations, costly hospitalizations, and diagnostic imaging exams^(22)^. In this regard, it is important to recognize the significance of a multidisciplinary approach that not only aims at pain relief but also considers the associated comorbidities, both physical and mental. This emphasizes the need for more integrated care strategies for workers with chronic pain, considering overall health and not just musculoskeletal pain in isolation.

Although the differences in pain between the sexes in the present study did not alter the comorbidity profile in either group, a systematic review^(23)^ concluded that patients with musculoskeletal disorders and chronic comorbidities present increased signs of central sensitization. These signs result in lower pain thresholds compared to individuals who only have musculoskeletal disorders with no comorbidities. This reinforces the importance of considering central sensitization in understanding chronic musculoskeletal pain in people with comorbidities, highlighting the complexity of this condition.

Another result demonstrating the complexity of understanding chronic musculoskeletal pain in relation to sex is that women presented weight within the expected normal standards, while men were more overweight. Excess weight can trigger or accentuate chronic musculoskeletal pain conditions, particularly in regions such as the knees, hips and back^(24)^, which in the present study were more prevalent among women. This therefore suggests that factors other than BMI, such as physiological, social and psychological aspects, may be playing a more important role in the pain characteristics in this group.

The frequency of continuous medication use in the present study was higher among women, with a higher consumption of analgesics and anticonvulsants. This result corroborates the literature^(25)^ and can be explained by the fact that women tend to use health services more than men^(26)^, as well as that women seek more alternatives to relieve symptoms when experiencing greater pain intensity. In addition, it is further confirmed given that there is a greater number of women who seek other therapeutic strategies to manage their pain through rehabilitation actions^(27)^.

However, although women professors in this study used more medications and various types of rehabilitation approaches, their pain intensity scores were higher, and their self-efficacy rates were lower than those of men professors. Additionally, although women receive more psychological care (psychotherapy) than men (according to this study), adherence to this type of treatment is particularly low considering its potential benefits for acquiring empowerment and developing self-management strategies for chronic musculoskeletal pain. A randomized controlled clinical trial found that the use of low-cost non-pharmacological approaches such as yoga, physical therapy, and pain education can improve cognitive aspects of pain, especially self-efficacy and pain catastrophizing, as well as pain intensity and physical function^(28)^.

Regarding the greater occurrence of breaks during the workday among men, it can be inferred that fewer breaks may justify a possible contribution from the increased physical and emotional overload of women, culminating in pain exacerbation in this group. Breaks are important moments for relaxation, allowing an interruption in the monotony of intellectual and mentally demanding activities in front of the computer and enabling to resume tasks with greater focus and efficiency. Moreover, they can contribute to muscle recovery and to relieve tensions which are common in individuals who work in static positions^(29)^.

The practical and theoretical implications of this study, based on comparisons between male and female professors, indicate the need for nursing teams in Brazilian public universities—particularly those focused on occupational health—to adopt a differentiated and personalized approach to managing chronic musculoskeletal pain in this population. Nursing professionals should lead holistic assessments that integrate biological, psychosocial, and occupational aspects, enabling the design of effective care plans and the implementation of gender-specific pain education protocols. Furthermore, nursing teams must collaborate with universities to promote actions that ensure continuous attention to improving teaching processes and working conditions and advocating for institutional policies that reduce gender disparities.

It is essential that universities strive to offer healthy environments that encompass adequate ergonomic aspects, respectful interpersonal relationships, pain education, teaching of self-management of pain conditions, encouraging self-care and autonomy, while also focusing on the reality of the data found in the different sexes with the objective of mitigating pain rates and equalizing conditions, optimizing productivity among professors and leaving aside the logic of intervention based exclusively on the biological process^(30)^.

Limitations of the study include the choice of the sample, which was only composed of professors from federal public universities, which may restrict understanding of the teaching-work scenario in Brazil, not considering other work contexts, such as private universities. Furthermore, in association analyses regarding the prevalence of moderate or intense chronic musculoskeletal pain between women and men, possible unmeasured confounding variables that are not investigated, such as those related to social, environmental or psychological factors, could explain changes in the associations between the adjusted variables.

As future perspectives, the use of a longitudinal study design to monitor and analyze outcomes over time can be mentioned. In addition, new research should carefully explore the intersectionality of sex and gender in chronic musculoskeletal pain to support interventions which not only improve the clinical outcomes of professors, but also their relationship with work regarding quality of life and productivity.

## CONCLUSION

Women professors experience more severe and widespread chronic musculoskeletal pain, use more medications and rehabilitation, and have lower self-efficacy and higher productivity loss compared"data-availability" to men. These findings highlight the need for gender-specific pain management strategies and improved workplace conditions to address these disparities.

## DATA AVAILABILITY

The entire dataset supporting the results of this study is available upon request to the corresponding author.
