# Supplementary material for: Differences in chronic musculoskeletal pain among male and female Brazilian university professors
Source: Rev Esc Enferm USP. 2026 Jan 23;60:e20250263. doi: 10.1590/1980-220X-REEUSP-2025-0263en (PMC12834387; doi:10.1590/1980-220X-REEUSP-2025-0263en)
Supplement: Translation

# Translation: Diferencias en el dolor musculoesquelético crónico entre profesores y profesoras universitarios brasileños*

**DOI:** 10.1590/1980-220X-REEUSP-2025-0263es

**Authors:** Mônica Jordão de Souza Pinto, Inmaculada Failde, Priscilla Hortense

**Affiliations:** 1 Universidade Federal de São Carlos, Programa de Pós-graduação em Enfermagem, São Carlos, SP, Brasil.; 2 Universidad de Cádiz, Medicina Preventiva y Salud Pública, Cadiz, Andalucía, España.; 3 Universidade Federal de São Carlos, Departamento de Enfermagem, São Carlos, SP, Brasil.

**Keywords:** Dolor crónico, Docentes, Caracteres Sexuales, Autoeficacia

## Abstract

**Objetivo:**

Comparar los aspectos sociodemográficos, clínicos, de dolor, laborales y de autoeficacia entre profesores universitarios brasileños de sexo masculino y femenino con dolor musculoesquelético crónico.

**Métodos:**

Este estudio transversal incluyó a 974 profesores universitarios con dolor musculoesquelético crónico. Datos para recolectar: encuesta electrónica realizada entre enero y mayo de 2023. Variables: características sociodemográficas y clínicas, intensidad del dolor, autoeficacia en el dolor crónico y pérdida de productividad laboral. Los análisis estadísticos utilizaron pruebas t, pruebas de Mann-Whitney y modelos de regresión.

**Resultados:**

Las mujeres informaron un 27 % más de áreas del cuerpo con dolor y un 10 % más de intensidad del dolor que los hombres. El uso continuo de analgésicos fue un 53 % más frecuente entre las mujeres, que también se sometieron a un 49 % más de tratamientos de rehabilitación. Las mujeres obtuvieron puntuaciones más bajas en la autoeficacia para el manejo del dolor y experimentaron una mayor pérdida de productividad. Las localizaciones de dolor más comunes en las mujeres fueron la región supraescapular, mientras que los hombres informaron de dolor lumbar. Las mujeres utilizaron más analgésicos y anticonvulsivos, y los hombres tuvieron tasas más altas de sobrepeso y obesidad.

**Conclusiones:**

Las mujeres experimentan un dolor musculoesquelético crónico más intenso y generalizado, utilizan más medicamentos y rehabilitación, y tienen una menor autoeficacia y una mayor pérdida de productividad en comparación con los hombres.

## INTRODUCCIÓN

El dolor crónico representa un importante reto para la salud pública y comprender su prevalencia contribuye al desarrollo de estrategias sanitarias adecuadas. Estudios recientes realizados en Estados Unidos y España evaluaron la prevalencia del dolor crónico, informando una prevalencia del 20,5 % entre los adultos en Estados Unidos^(1)^ y del 25,9 % entre los adultos y los adultos mayores en España^(2)^. En Brasil, la prevalencia del dolor crónico se identificó en una revisión sistemática con metaanálisis en la población adulta general, oscilando entre el 23,02 % y el 41,4 %^(3)^.

Entre los diferentes tipos de dolor crónico, el dolor musculoesquelético crónico destaca por su alta prevalencia, lo que lo convierte en una importante preocupación sanitaria para investigadores, médicos y empleadores de todo el mundo. Un estudio realizado en varios países europeos con 61 157 personas de la población general mayor de 50 años mostró que la prevalencia del dolor musculoesquelético crónico era del 35,7 %, oscilando entre el 18,6 % en Suiza y el 45,6 % en Francia^(4)^. La prevalencia en los países de ingresos bajos y medios fue del 26 %, 39 % y 86 % en adultos, adultos mayores y trabajadores, respectivamente^(5)^.

El sexo constituye un factor relevante en cuanto a las disparidades en la prevalencia y las características de las personas con dolor musculoesquelético crónico. Las tasas de este tipo de dolor son más altas entre las mujeres ^(2,4,6-10)^, que presentan mayor intensidad, frecuencia, número de zonas del cuerpo afectadas y duración en comparación con los hombres ^(6-7,9-10)^. Además, las mujeres también se ven más afectadas por enfermedades que causan dolor, como la fibromialgia, los trastornos temporomandibulares, la osteoartritis y la artritis reumatoide, así como el dolor cervical y el dolor lumbar^(6,10)^.

El mundo laboral es un ámbito destacado para investigar las disparidades sexuales en el dolor musculoesquelético crónico, dada su prevalencia entre los trabajadores^(5)^. Este contexto es aún más relevante para la investigación, ya que permite la comparación entre hombres y mujeres, teniendo en cuenta que, además del trabajo formal, las mujeres acumulan otras funciones, lo que aumenta su sobrecarga y su exposición al riesgo de dolor musculoesquelético crónico.

Los docentes están expuestos a importantes factores de riesgo para el desarrollo de trastornos musculoesqueléticos y, en consecuencia, de dolor musculoesquelético crónico. Estos factores están relacionados con la naturaleza del trabajo físico docente, que incluye estar de pie y/o sentado durante largos periodos de tiempo en posturas inadecuadas, inclinarse sobre los escritorios para leer o evaluar el trabajo de los estudiantes, y cargar y transportar libros pesados o equipo para el aula^(11)^. En Brasil, los profesores universitarios representan un importante grupo de trabajadores con una elevada carga de trabajo, que incluye la docencia, la investigación, la extensión y la gestión. Por lo tanto, identificar las demandas de salud en este grupo para conocer mejor las diferencias sexuales en el dolor musculoesquelético crónico puede proporcionar conocimientos para crear protocolos de tratamiento adaptados a las necesidades identificadas^(6-7,10)^.

El análisis de estas diferencias, tanto en términos de aspectos clínicos como de los relacionados con el trabajo y el manejo del dolor, puede proporcionar una comprensión integral y fundamental para esbozar el perfil de implicación y ser una evidencia crucial para que los médicos y las instituciones empleadoras desarrollen estrategias de manejo específicas. Así, el presente estudio contribuye al conocimiento derivado de la investigación en seres humanos con dolor crónico, ya que se diferencia de muchos otros que han evaluado las diferencias de género en el dolor en modelos animales o inducido en voluntarios sanos^(12)^.

Por lo tanto, este estudio tiene como objetivo comparar a hombres y mujeres en cuanto a aspectos sociodemográficos, clínicos, de dolor, trabajo y autoeficacia en una muestra de profesores de universidades públicas federales brasileñas que presentan dolor musculoesquelético crónico.

## MÉTODOS

### Diseño

Este estudio es una investigación analítica y transversal^(13)^ guiada por las directrices STROBE.

### Lugar

El estudio se llevó a cabo en las 68 universidades federales brasileñas activas.

### Definición de la Muestra

Para reclutar a los participantes, utilizamos un enfoque de muestreo por conveniencia. Se enviaron invitaciones mensuales para participar a aproximadamente 9000 correos electrónicos seleccionados al azar en los sitios web de las universidades, incluidos los correos electrónicos personales de los profesores y los correos electrónicos de las secretarías con solicitudes para difundir la encuesta. Estas invitaciones no estaban dirigidas específicamente a personas con dolor musculoesquelético, sino a la población universitaria en general. Para alcanzar los objetivos de este estudio, solo se incluyeron los profesores que respondieron afirmativamente a la pregunta de si habían tenido dolor musculoesquelético durante 6 meses o más. Para facilitar la respuesta, se adjuntó el concepto de dolor musculoesquelético a la pregunta y se pidió a los participantes que indicaran durante cuánto tiempo habían tenido dolor, de modo que solo pudimos incluir a aquellos que informaron de dolor durante 6 meses o más. Obtuvimos 1436 respuestas y se excluyó a 462 profesores porque no informaron de dolor musculoesquelético crónico.

### Población y Criterios de Selección

Los criterios de inclusión fueron ser profesor, tener un régimen de empleo estatutario (funcionario con estabilidad laboral) y declarar haber tenido dolor musculoesquelético crónico durante seis meses o más. Se excluyó a los profesores nombrados para su cargo menos de un año antes de la fecha de recopilación de datos, a los que habían estado ausentes de sus actividades laborales en las dos últimas semanas tras recibir la invitación, a los jubilados y a los que padecían dolor oncológico crónico.

### Recopilación de Datos y Variables Estudiadas

La recopilación de datos se realizó de forma virtual (encuesta electrónica) entre enero y mayo de 2023. Las variables investigadas para la comparación entre sexos fueron autoinformadas e incluyeron la edad; el índice de masa corporal (IMC); número de regiones del cuerpo con dolor; comorbilidades; localización, intensidad y frecuencia del dolor; tratamientos farmacológicos y no farmacológicos para el dolor; autoeficacia; datos laborales (otro trabajo, horas extras - número de horas trabajadas más allá del contrato de trabajo, satisfacción); y pérdida de productividad en el trabajo. Es importante destacar que este estudio solo se centró en el sexo como variable biológica y no investigó la influencia del género, que se refiere a los roles, comportamientos e identidades sociales.

La intensidad del dolor se midió utilizando una escala numérica de 11 puntos y se clasificó de la siguiente manera: un valor de cero representa ausencia de dolor, un rango de 1 a 3 indica dolor leve, un rango de 4 a 6 indica dolor moderado y un rango de 7 a 10 indica dolor intenso^(14)^. y los tratamientos evaluados incluyeron medidas farmacológicas (nombre de los medicamentos utilizados, que se clasificaron por clase farmacológica) y medidas no farmacológicas (rehabilitación), incluyendo Pilates, natación, aeróbicos acuáticos, acupuntura, meditación, fisioterapia, reeducación postural general, psicoterapia, control nutricional, ejercicios físicos con un educador físico, monitoreo con un médico, grupo de apoyo para el dolor y otros, además del tiempo de finalización en meses.

Posteriormente, se utilizó la Escala de Autoeficacia para el Dolor Crónico (Chronic Pain Self-Efficacy Scale - CPSS), validada en Brasil^(15)^, para medir la autoeficacia para el dolor crónico, con 22 preguntas distribuidas en tres ámbitos: autoeficacia para el manejo del dolor, autoeficacia para el funcionamiento físico y autoeficacia para hacer frente a los síntomas. Las puntuaciones oscilan entre 30 y 300, y cuanto mayor es la puntuación, mayor es el nivel de autoeficacia para el dolor crónico. La pérdida de productividad se estimó utilizando el Cuestionario de Limitaciones Laborales (Work Limitations Questionnaire - WLQ-25), validado en Brasil^(16)^, y se firmó un acuerdo de confidencialidad.

### Análisis de Datos y Tratamiento

Los datos se describieron mediante frecuencias absolutas y porcentuales (variables cualitativas) y mediante medidas de tendencia central y dispersión (media, desviación estándar, mínimo, mediana y máximo) para las variables cuantitativas, estratificadas por sexo.

Las comparaciones de variables cuantitativas continuas entre sexos se realizaron mediante la prueba t de Student o la prueba de Mann-Whitney, según la normalidad (verificada mediante histograma y prueba de Kolmogorov-Smirnov). Se utilizó un modelo de regresión binomial negativa y una función de enlace logarítmica para las variables cuantitativas discretas (número de regiones con dolor, intensidad media del dolor y número de medicamentos en uso continuo), calculando el aumento o la reducción relativa de la media mediante AR(>β) = [exp(>β) – 1]*100 %. Las variables categóricas con más de dos niveles de respuesta se analizaron mediante la prueba de chi cuadrado, mientras que las variables binarias se analizaron mediante un modelo de regresión log-binomial con estimación de la razón de prevalencia. Todos los análisis se realizaron utilizando el software SAS 9.4 con un nivel de significación del 5 %, y los gráficos se generaron utilizando el software R Studio (versión 2023.12.0.369).

### Aspectos Éticos

El estudio fue aprobado por el Comité de Ética en Investigación de la Universidad Federal de São Carlos (dictamen n.º 5.741.976) y todos los participantes aceptaron participar firmando el formulario de consentimiento informado en formato digital.

## RESULTADOS

El estudio incluyó a 974 profesores universitarios con dolor musculoesquelético crónico de todas las regiones de Brasil, de los cuales 622 eran mujeres (63,8 %) y 352 (36,1 %) eran hombres, con poca variación en la edad media. La frecuencia de comorbilidades fue similar entre ambos sexos, y los hombres presentaron una mayor tasa de sobrepeso y obesidad en relación con el IMC (Tabla 1).

**Tabla 1:** Comparación entre profesores universitarios federales de sexo masculino y femenino con dolor musculoesquelético crónico en cuanto a edad, características clínicas, dolor y su manejo, y escala de autoeficacia para el dolor crónico (CPSS) – Brasil, 2023.

Variable	Mujeres (n = 622)	Hombres (n = 352)	Valor p	Efecto (IC del 95 %) Mujeres frente a hombres
**Edad**			0,75^1^	DE: 0,19 (–0,99; 1,37)
Media (DE)	48,11 (8,93)	47,92 (9,1)		
Mediana (mín.-máx.)	47 (29–73)	47 (21–74)		
**Comorbilidades**				
No	378 (60,77 %)	209 (59,38 %)		
Sí	244 (39,23 %)	143 (40,63 %)	0,67^4^	PR: 0,97 (0,82; 1,13)
**IMC**			<0,01 ^3^	
Bajo peso	11 (1,77 %)	1 (0,28 %)		
Peso normal	283 (45,5 %)	106 (30,11 %)		
Sobrepeso	210 (33,76 %)	161 (45,74 %)		
Obesidad	118 (18,97 %)	84 (23,86 %)		
**Número de zonas del cuerpo en las que se siente dolor**			<0,01 ^5^	RD: 1,27 (1,17; 1,39)
Media (DE)	4,38 (2,98)	3,44 (2,31)		
Mediana (mín.-máx.)	4 (0–17)	3 (0–17)		
**Intensidad del dolor**			**<0,01** ^5^	RD: 1,10 (1,04; 1,17)
Media (DE)	5,97 (2,13)	5,41 (2,02)		
Mediana (mín.-máx.)	6 (0-10)	6 (0–10)		
**Frecuencia semanal del dolor**			0,01^3^	
1 vez	38 (6,11 %)	22 (6,25 %)		
2 veces	70 (11,25 %)	50 (14,2 %)		
3 veces	81 (13,02 %)	69 (19,6 %)		
4 veces	72 (11,58 %)	42 (11,93 %)		
5 veces	98 (15,76 %)	44 (12,5 %)		
6 veces	39 (6,27 %)	9 (2,56 %)		
7 veces	224 (36,01 %)	116 (32,95 %)		
**Uso continuo de analgésicos**				
No	382 (61,41 %)	263 (74,72 %)		
Sí	240 (38,59 %)	89 (25,28 %)	<0,01^4^	PR: 1,53 (1,24; 1,87)
**Número de medicamentos utilizados de forma continua para el dolor**			**<0,01** ^5^	RD: 1,68 (1,32; 2,14)
Media (DE)	0,63 (0,97)	0,38 (0,75)		
Mediana (mín.-máx.)	0 (0–5)	0 (0–4)		
**Uso intermitente de analgésicos**				
No	203 (32,64 %)	123 (34,94 %)		
Sí	419 (67,36 %)	229 (65,06 %)	0,47^4^	PR: 1,04 (0,94; 1,14)
**Rehabilitación para el dolor**				
No	265 (42,6 %)	216 (61,36 %)		
Sí	357 (57,4 %)	136 (38,64 %)	**<0,01** ^4^	PR: 1,49 (1,28; 1,72)
**Autoeficacia para el manejo del dolor**			**<0,01** ^1^	ED: –4,04 (–7,02; -1,06)
Media (DE)	57,86 (22,67)	61,9 (22,97)		
Mediana (mín.-máx.)	58 (10–100)	64 (10–100)		
**Autoeficacia para el funcionamiento físico**			<0,01^2^	
Media (DE)	70,34 (26,06)	75,06 (26,71)		
Mediana (mín.-máx.)	76,67 (0–100)	80 (0–100)		
**Autoeficacia para hacer frente a los síntomas**			**<0,01** ^1^	ED: –4,83 (–7,65; –2,02)
Media (DE)	57,03 (21,25)	61,86 (21,97)		
Mediana (mín.-máx.)	57,5 (0–100)	63,75 (10–100)		
**Puntuación total de autoeficacia**			**<0,01** ^1^	ED: –13,59 (–21,9; –5,28)
Media (DE)	185,22 (62,82)	198,81 (64,57)		
Mediana (mín.-máx.)	193,53 (29,86-300)	209,42 (30–300)		

^1^ Prueba t de Student; ED: diferencia estimada.

^2^ Prueba de Mann-Whitney.

^3^ Prueba dechi cuadrado.

^4^ Regresión log-binomial; PR: Razón de prevalencia.

^5^ Regresión binomial negativa con función de enlace logarítmica; RD: diferencia relativa.

En cuanto al dolor, el número de regiones corporales con dolor en las mujeres es un 27 % mayor en comparación con los hombres, y ellas experimentan un dolor un 10 % más intenso que ellos (Tabla 1). La región más frecuentemente reportada entre las docentes es la región supraescapular, mientras que para los hombres es la región lumbar (Tabla 2). Además, otras localizaciones de dolor frecuentemente reportadas por ambos sexos fueron la región cervical y los hombros.

**Tabla 2:** Comparación entre profesores universitarios federales de sexo masculino y femenino con dolor musculoesquelético crónico en cuanto a la localización corporal) – Brasil, 2023

Localización del dolor	Mujeres (n = 622)	Hombres (n = 352)	Valor p^1^	PR (IC95 %)
Suprascapular	370 (59,49 %)	130 (36,93 %)	<0,01	1,61 (1,38; 1,87)
Parte inferior de la espalda	325 (52,25 %)	178 (50,57 %)	0,62	1,03 (0,91; 1,17)
Cervical	273 (43,89 %)	118 (33,52 %)	<0,01	1,31 (1,1; 1,55)
Hombros	265 (42,6 %)	114 (32,39 %)	<0,01	1,32 (1,1; 1,57)
Cadera	232 (37,3 %)	89 (25,28 %)	<0,01	1,48 (1,2; 1,81)
Rodillas	173 (27,81 %)	101 (28,69 %)	0,77	0,97 (0,79; 1,19)
Muñecas	154 (24,76 %)	53 (15,06 %)	<0,01	1,64 (1,24; 2,18)
Parte superior de la espalda	143 (22,99 %)	72 (20,45 %)	0,36	1,12 (0,87; 1,45)
Manos	135 (21,7 %)	44 (12,5 %)	<0,01	1,74 (1,27; 2,38)
Pies	117 (18,81 %)	53 (15,06 %)	0,14	1,25 (0,93; 1,68)
Parte media de la espalda	113 (18,17 %)	69 (19,6 %)	0,58	0,93 (0,71; 1,21)
Codos	99 (15,92 %)	44 (12,5 %)	<0,01	1,32 (1,1; 1,57)
Tobillos	90 (14,47 %)	38 (10,8 %)	0,11	1,34 (0,94; 1,91)
Antebrazos	69 (11,09 %)	18 (5,11 %)	<0,01	2,17 (1,31; 3,58)
Muslos	60 (9,65 %)	38 (10,8 %)	0,57	0,89 (0,61; 1,31)
Piernas	54 (8,68 %)	34 (9,66 %)	0,61	0,9 (0,6; 1,35)
Brazos	52 (8,36 %)	17 (4,83 %)	0,04	1,73 (1,02; 2,95)

^1^ Regresión log-binomial;

PR: Razón de prevalencia.

La frecuencia del uso continuo de analgésicos en las mujeres es un 53 % mayor que en los hombres (Tabla 1) y los medicamentos más utilizados por ambos sexos son los antiinflamatorios y los relajantes musculares. El uso de analgésicos y anticonvulsivos es mayor entre las mujeres (Tabla 3).

**Tabla 3:** Comparación entre profesores universitarios federales hombres y mujeres en cuanto a las medidas farmacológicas y no farmacológicas (rehabilitación) utilizadas en el tratamiento del dolor – Brasil, 2023.

Tratamientos para el dolor	Mujeres (n = 613)	Hombres (n = 349)	Valor p	PR (IC 95 %) Mujeres frente a hombres
**Medicamentos de uso continuo**				
Antiinflamatorios	103 (16,8 %)	47 (13,47 %)	0,17^1^	1,25 (0,91; 1,72)
Relajantes musculares	90 (14,68 %)	27 (7,74 %)	<0,01^1^	1,9 (1,26; 2,86)
Analgésicos	56 (9,14 %)	17 (4,87 %)	0,02^1^	1,88 (1,11; 3,18)
Anticonvulsivos	38 (6,2 %)	14 (4,01 %)	0,15^1^	1,55 (0,85; 2,81)
Antidepresivos	35 (5,71 %)	4 (1,15 %)	<0,01^1^	4,98 (1,79; 13,9)
Analgésicos narcóticos	33 (5,38 %)	11 (3,15 %)	0,12^1^	1,71 (0,87; 3,34)
Otros	17 (2,77 %)	6 (1,72 %)	0,31^1^	1,61 (0,64; 4,05)
Glucocorticoides sistémicos	14 (2,28 %)	5 (1,43 %)	0,37^1^	1,59 (0,58; 4,39)
**Rehabilitación**				
Fisioterapia	229 (36,82 %)	83 (23,58 %)	<0,01^1^	1,56 (1,26; 1,93)
Pilates	211 (33,92 %)	65 (18,47 %)	<0,01^1^	1,84 (1,44; 2,35)
Seguimiento con un médico	117 (18,81 %)	30 (8,52 %)	<0,01^1^	2,21 (1,51; 3,22)
Acupuntura	112 (18,01 %)	31 (8,81 %)	<0,01^1^	2,04 (1,4; 298)
Ejercicios físicos con un educador físico	89 (14,31 %)	30 (8,52 %)	<0,01^1^	1,68 (1,13; 2,49)
Otros	62 (9,97 %)	32 (9,09 %)	0,66^1^	1,1 (0,73; 1,65)
Reeducación postural general	60 (9,65 %)	9 (2,56 %)	<0,01^1^	3,77 (1,9; 7,51)
Psicoterapia	53 (8,52 %)	14 (3,98 %)	<0,01^1^	2,14 (1,21; 3,8)
Control nutricional	46 (7,4 %)	16 (4,55 %)	0,08^1^	1,63 (0,94; 2,83)
Aeróbicos acuáticos	40 (6,43 %)	10 (2,84 %)	0,02^1^	2,26 (1,15; 4,47)
Natación	36 (5,79 %)	26 (7,39 %)	0,33^1^	0,78 (0,48; 1,28)
Meditación	35 (5,63 %)	12 (3,41 %)	0,13^1^	1,65 (0,87; 3,14)
Grupo de apoyo para el dolor	3 (0,48 %)	0 (0 %)	0,56^3^	–
**Número de actividades de rehabilitación**			<0,01^2^	–
Media (DE)	1,76 (1,96)	1,02 (1,62)		
Mediana (mín.-máx.)	1 (0–10)	0 (0–10)		

^1^ Regresión log-binomial;

PR: Razón de prevalencia.

^2^ Prueba de Mann-Whitney.

^3^ Prueba exacta de Fisher.

La frecuencia de la rehabilitación para el dolor es un 49 % mayor entre las mujeres, que se someten a una media de 1,76 tipos de rehabilitación, mientras que los hombres se someten a 1,02 (Tabla 1). La fisioterapia es el tipo más solicitado, seguido del Pilates. Otras intervenciones frecuentes fueron el seguimiento médico, la acupuntura y los ejercicios físicos con un profesional de la educación física (Tabla 3).

Las mujeres obtuvieron una puntuación 13,59 puntos inferior a la de los hombres en la puntuación total de autoeficacia. Además, las mujeres obtuvieron una puntuación 4 y 4,8 puntos inferior a la de los hombres en los dominios de autoeficacia en el manejo del dolor y el afrontamiento de los síntomas, respectivamente. No hubo diferencias estadísticamente significativas entre los sexos en el número de horas trabajadas más allá de la jornada laboral semanal. Las docentes tomaron un 16 % menos de descansos durante la jornada laboral en comparación con los profesores, además de presentar una pérdida media de productividad en el trabajo un 1,22 % mayor (Tabla 4).

**Tabla 4:** Comparación entre profesores universitarios federales de sexo masculino y femenino con dolor musculoesquelético crónico en relación con los datos laborales y la pérdida de productividad en el trabajo – Brasil, 2023.

Variable	Mujeres (n = 622)	Hombres (n = 352)	Valor p	Efecto (IC del 95 %) Mujeres frente a hombres
**Horas de trabajo superiores a las horas de trabajo semanales**			0,183	PR: 1,05 (0,98; 1,14)
No	145 (23,31 %)	96 (27,27 %)		
Sí	477 (76,69 %)	256 (72,73 %)		
**Número de horas adicionales trabajadas por semana**			0,472	
Menos de 1 hora/semana	2 (0,42 %)	0 (0 %)		
De 1 a 5 horas a la semana	164 (34,38 %)	90 (35,16 %)		
De 6 a 10 horas/semana	166 (34,8 %)	98 (38,28 %)		
Más de 10 horas/semana	145 (30,4 %)	68 (26,56 %)		
**Descanso durante la jornada laboral**			**<0,014**	PR: 0,84 (0,76; 0,93)
No	261 (41,96 %)	109 (30,97 %)		
Sí	361 (58,04 %)	243 (69,03 %)		
**Satisfacción laboral**			0,743	PR: 0,99 (0,95; 1,03)
No	57 (9,18 %)	30 (8,55 %)		
Sí	564 (90,82 %)	321 (91,45 %)		
**Pérdida de productividad en el trabajo (porcentaje)**			**<0,011**	DE: 1,22 (0,57; 1,88)
Media (DE)	8,27 (5,03)	7,05 (4,9)		
Mediana (Mín.-Máx.)	7,51 (0-24,08)	6,2 (0-21,84)		

^1^ Prueba t de Student; ED: diferencia estimada.

^2^ Prueba de chi cuadrado.

^3^ Regresión log-binomial. PR: Razón de prevalencia.

Se utilizó un modelo de regresión log-binomial no ajustado con cada variable independiente analizada individualmente para las variables independientes cuantitativas, y se estimó la razón de prevalencia para cada aumento de 1 unidad en la variable independiente. En las mujeres, el aumento de la edad, el número de regiones del cuerpo con dolor, un mayor número de medicamentos para el dolor continuos, la insatisfacción con la enseñanza y una mayor pérdida de productividad se asocian con una mayor prevalencia de dolor moderado o intenso (puntuación ≥ 4).

Además, una puntuación más alta en autoeficacia se asocia con una menor prevalencia de dolor moderado o intenso (puntuación ≥ 4). En los hombres, una mayor pérdida de productividad se asocia con una mayor prevalencia de dolor moderado o intenso (en promedio, por cada aumento de 1 punto en la pérdida de productividad, la prevalencia de dolor moderado o intenso aumenta en un 1,3 %) y una puntuación más alta en autoeficacia se asocia con una menor prevalencia de dolor moderado o intenso. Sin embargo, en los análisis ajustados con todas las variables, no se encontraron asociaciones. Del mismo modo, no se encontraron asociaciones en un segundo análisis ajustado que incluía como variables independientes solo aquellas con un p-valor <0,20 en los análisis brutos.

## DISCUSIÓN

Los resultados de este estudio muestran las diferencias por sexo en el dolor musculoesquelético crónico entre los profesores universitarios, y estas diferencias se evidencian en la mayor prevalencia de este tipo de dolor entre las mujeres. Las mujeres también presentaron una mayor intensidad del dolor y un mayor número de **áreas** afectadas, una mayor frecuencia de uso de medicamentos y tratamientos de rehabilitación para el dolor, un menor **índice** de autoeficacia general y de autoeficacia para el manejo del dolor y el afrontamiento de los síntomas, menos descansos durante la jornada laboral y una mayor pérdida de productividad en el trabajo en comparación con los hombres.

Los resultados del presente estudio muestran lo que otros estudios han encontrado con respecto a la mayor prevalencia del dolor crónico entre las mujeres; dichos estudios han demostrado que los aspectos genéticos, anatómicos, fisiológicos, neuronales, hormonales, psicológicos, sociales y culturales influyen y causan diferencias en la percepción, modulación y expresión del dolor entre las mujeres y los hombres^(7,10)^, y que dichas diferencias incluyen la comunicación, las actitudes y el comportamiento ante el dolor.

Cabe destacar que las diferencias por sexo en la percepción del dolor se ven fuertemente influidas por factores psicosociales y culturales que pueden actuar desde el nacimiento, pasando por la infancia, hasta la edad adulta^(17-18)^. Estos factores están posiblemente relacionados con la forma en que la sociedad trata el dolor en los niños y las niñas, así como en las mujeres y los hombres. Las variables psicosociales desempeñan un papel importante en el desarrollo y el mantenimiento del dolor, además de modular los resultados del tratamiento^(17)^. Las variables psicosociales que han demostrado desempeñar este papel son el afecto negativo, los traumas infantiles y el apoyo social, así como la catastrofización del dolor, la autoeficacia para lidiar con el dolor y la capacidad de afrontarlo^(18)^.

Los diversos roles que asumen las mujeres en la sociedad actual, las responsabilidades acumuladas, las exigencias de un desempeño óptimo en los roles de esposa, madre, administradora del hogar y trabajadora son aspectos importantes que configuran la expresión del dolor y modulan el estímulo nociceptivo, lo que explica los resultados de mayor prevalencia, intensidad y número de áreas del cuerpo con dolor. Además, existe la posible falta de apoyo social a las mujeres en sus roles, lo que genera una sobrecarga física y emocional y contribuye al empeoramiento del dolor^(18)^.

Además de estas cuestiones, es importante destacar que la gran mayoría de los docentes de las universidades públicas de Brasil tienen un horario de trabajo exclusivo de 40 horas semanales y son responsables de las actividades de docencia, investigación, extensión y gestión universitaria. Sumado a todas las demás responsabilidades que asumen las mujeres, este horario puede acarrear varias consecuencias, entre ellas el agotamiento físico y mental, lo que acentúa y agrava la condición de dolor crónico y otras afecciones de salud^(19)^.

En cuanto a la localización del dolor, las regiones cervical y del hombro fueron zonas comunes de dolor en ambos sexos. La literatura indica que estas zonas se señalan con frecuencia como foco de dolor en los trabajadores que realizan sus actividades frente a computadoras y dispositivos electrónicos durante horas y horas, y a menudo sin los ajustes ergonómicos adecuados de los puestos de trabajo necesarios para prevenir el dolor ^(20)^. Esto se aplica especialmente a la categoría de profesores en Brasil, que en su mayoría realizan sus tareas en posición sentada, como preparar clases para estudiantes de licenciatura y posgrado, corregir becas de investigación de licenciatura, maestría y doctorado, leer y realizar actividades de investigación científica, gestionar a los estudiantes, entre otras.

La frecuencia de las comorbilidades fue similar entre ambos sexos, lo que sugiere que, a pesar de las disparidades encontradas en la intensidad y el número de regiones de dolor, las mujeres y los hombres presentaban un estado de salud similar en relación con otras condiciones clínicas. Este resultado es relevante, ya que demuestra que las variaciones en el dolor musculoesquelético crónico no están asociadas con un mayor número de comorbilidades entre los grupos. Sin embargo, es importante destacar que, aunque los profesores de ambos sexos tienen una edad media de 48 años, alrededor del 40 % de cada grupo presenta comorbilidades. Este índice es preocupante si se compara con la población brasileña de entre 45 y 54 años sin dolor, cuya prevalencia de morbilidad es del 34,7 % para la hipertensión, del 10,4 % para la diabetes mellitus y del 11,8 % para la depresión^(21)^.

Además, las personas con dolor crónico tienen una mayor prevalencia de comorbilidades, y el dolor puede actuar como factor de riesgo para el empeoramiento de estas afecciones, creando un círculo vicioso que aumenta la carga de la enfermedad y da lugar a tasas significativamente más altas de consultas médicas, hospitalizaciones costosas y exámenes de diagnóstico por imagen^(22)^. En este sentido, es importante reconocer la importancia de un enfoque multidisciplinario que no solo tenga como objetivo el alivio del dolor, sino que también tenga en cuenta las comorbilidades asociadas, tanto físicas como mentales. Esto pone de relieve la necesidad de estrategias de atención más integradas para los trabajadores con dolor crónico, teniendo en cuenta la salud general y no solo el dolor musculoesquelético de forma aislada.

Aunque las diferencias en el dolor entre los sexos en el presente estudio no alteraron el perfil de comorbilidad en ninguno de los dos grupos, una revisión sistemática^(23)^ concluyó que los pacientes con trastornos musculoesqueléticos y comorbilidades crónicas presentan un aumento de los signos de sensibilización central. Estos signos dan lugar a umbrales de dolor más bajos en comparación con las personas que solo padecen trastornos musculoesqueléticos sin comorbilidades. Esto refuerza la importancia de tener en cuenta la sensibilización central para comprender el dolor musculoesquelético crónico en personas con comorbilidades, lo que pone de relieve la complejidad de esta afección.

Otro resultado que demuestra la complejidad de comprender el dolor musculoesquelético crónico en relación con el sexo es que las mujeres presentaban un peso dentro de los estándares normales esperados, mientras que los hombres tenían más sobrepeso. El exceso de peso puede desencadenar o acentuar las afecciones de dolor musculoesquelético crónico, especialmente en regiones como las rodillas, las caderas y la espalda^(24)^, que en el presente estudio fueron más prevalentes entre las mujeres. Por lo tanto, esto sugiere que otros factores distintos del IMC, como los aspectos fisiológicos, sociales y psicológicos, pueden estar desempeñando un papel más importante en las características del dolor en este grupo.

La frecuencia del uso continuo de medicamentos en el presente estudio fue mayor entre las mujeres, con un mayor consumo de analgésicos y anticonvulsivos. Este resultado corrobora la literatura^(25)^ y puede explicarse por el hecho de que las mujeres tienden a utilizar más los servicios de salud que los hombres^(26)^, así como por el hecho de que las mujeres buscan más alternativas para aliviar los síntomas cuando experimentan una mayor intensidad del dolor. Además, se confirma aún más dado que hay un mayor número de mujeres que buscan otras estrategias terapéuticas para manejar su dolor a través de acciones de rehabilitación^(27)^.

Sin embargo, aunque las docentes de este estudio utilizaron más medicamentos y diversos tipos de enfoques de rehabilitación, sus puntuaciones de intensidad del dolor fueron más altas y sus índices de autoeficacia más bajos que los de los profesores. Además, aunque las mujeres reciben más atención psicológica (psicoterapia) que los hombres (según este estudio), la adherencia a este tipo de tratamiento es particularmente baja teniendo en cuenta sus posibles beneficios para adquirir empoderamiento y desarrollar estrategias de autocontrol del dolor musculoesquelético crónico. Un ensayo clínico aleatorizado y controlado reveló que el uso de enfoques no farmacológicos de bajo costo, como el yoga, la fisioterapia y la educación sobre el dolor, puede mejorar los aspectos cognitivos del dolor, especialmente la autoeficacia y la catastrofización del dolor, así como la intensidad del dolor y la función física^(28)^.

En cuanto a la mayor incidencia de descansos durante la jornada laboral entre los hombres, se puede deducir que un menor número de descansos puede justificar una posible contribución del aumento de la sobrecarga física y emocional de las mujeres, que culmina en la exacerbación del dolor en este grupo. Los descansos son momentos importantes para la relajación, ya que permiten interrumpir la monotonía de las actividades intelectuales y mentalmente exigentes frente a la computadora y permiten reanudar las tareas con mayor concentración y eficiencia. Además, pueden contribuir a la recuperación muscular y a aliviar las tensiones que son comunes en las personas que trabajan en posiciones estáticas^(29)^.

Las implicaciones prácticas y teóricas de este estudio, basadas en comparaciones entre profesores y docentes, indican la necesidad de que los equipos de enfermería de las universidades públicas brasileñas, en particular los centrados en la salud ocupacional, adopten un enfoque diferenciado y personalizado para el manejo del dolor musculoesquelético crónico en esta población. Los profesionales de enfermería deben realizar evaluaciones holísticas que integren aspectos biológicos, psicosociales y ocupacionales, lo que permitirá diseñar planes de atención eficaces y aplicar protocolos de educación sobre el dolor específicos para cada género. Además, los equipos de enfermería deben colaborar con las universidades para promover acciones que garanticen una atención continua a la mejora de los procesos docentes y las condiciones de trabajo, y abogar por políticas institucionales que reduzcan las disparidades de género.

Es esencial que las universidades se esfuercen por ofrecer entornos saludables que abarquen aspectos ergonómicos adecuados, relaciones interpersonales respetuosas, educación sobre el dolor, enseñanza del autocontrol de las afecciones dolorosas, fomento del autocuidado y la autonomía, al tiempo que se centran en la realidad de los datos encontrados en los diferentes sexos con el objetivo de mitigar las tasas de dolor e igualar las condiciones, optimizar la productividad entre los profesores y dejar de lado la lógica de intervención basada exclusivamente en el proceso biológico^(30)^.

Las limitaciones del estudio incluyen la elección de la muestra, que solo estaba compuesta por profesores de universidades públicas federales, lo que puede restringir la comprensión del escenario docente-laboral en Brasil, sin considerar otros contextos laborales, como las universidades privadas. Además, en los análisis de asociación relativos a la prevalencia del dolor musculoesquelético crónico moderado o intenso entre mujeres y hombres, las posibles variables de confusión no medidas que no se investigan, como las relacionadas con factores sociales, ambientales o psicológicos, podrían explicar los cambios en las asociaciones entre las variables ajustadas.

Como perspectivas futuras, cabe mencionar el uso de un diseño de estudio longitudinal para monitorear y analizar los resultados a lo largo del tiempo. Además, las nuevas investigaciones deberían explorar cuidadosamente la interseccionalidad del sexo y el género en el dolor musculoesquelético crónico para apoyar intervenciones que no solo mejoren los resultados clínicos de los profesores, sino también su relación con el trabajo en lo que respecta a la calidad de vida y la productividad.

## CONCLUSIÓN

Las docentes experimentan un dolor musculoesquelético crónico más intenso y generalizado, utilizan más medicamentos y rehabilitación, y tienen una menor autoeficacia y una mayor pérdida de productividad en comparación con los hombres. Estos hallazgos ponen de relieve la necesidad de estrategias de manejo del dolor específicas para cada género y de mejorar las condiciones del lugar de trabajo para abordar estas disparidades.

## DISPONIBILIDAD DE DATOS

El conjunto de datos que respaldan las conclusiones de este estudio está disponible previa solicitud al autor correspondiente.
